# Late BK Nephropathy 15 years post kidney transplant following chemotherapy: A case report

**DOI:** 10.1016/j.idcr.2025.e02340

**Published:** 2025-08-08

**Authors:** Anum Hamiduzzaman, Jean Hou, Tram Higgins, Pryce Gaynor, Abhirami Shankar, Erik L. Lum

**Affiliations:** a Division of Nephrology, Department of Medicine, David Geffen School of Medicine at UCLA, Los Angeles, CA 90095, USA; b Department of Pathology and Laboratory Medicine, Cedars Sinai Medical Center, Los Angeles, CA 90048, USA; c Department of Transplant Services, Kidney and Pancreas Transplant. UCLA. Los Angeles, CA 90095, USA; d Division of Infectious Disease, Department of Medicine, David Geffen School of Medicine at UCLA, Los Angeles, CA 90095, USA; e Harbor UCLA Medical Center Division of Nephrology, Los Angeles, CA 90502, USA

**Keywords:** BK nephropathy, Multiple Myeloma, Kidney Transplant

## Abstract

BK Polyomavirus (BKPyV) is an important risk factor for premature graft loss following kidney transplant. Current practice guidelines recommend screening for BK virus DNA for 2 years after kidney transplant as the risk of BK Polyomavirus associated nephropathy (BKPyVAN) wanes over time. This case report presents a unique scenario of late onset BKPyVAN 15 years following kidney transplant precipitated by administration of chemotherapy to treat multiple myeloma. A woman in her 60 s with no prior history of BKPyV was diagnosed with multiple myeloma 14 years after kidney transplant. Her immunosuppression was tapered to tacrolimus and prednisone before undergoing chemotherapy treatment for her multiple myeloma. Although the chemotherapy was effective in achieving remission of her myeloma, she developed worsening renal dysfunction without proteinuria. Kidney biopsy revealed positive SV40 staining and subsequent BK DNA serum PCR was found to be 236,000 copies/mL suggestive of BKPyVAN. Although incredibly rare, late onset BKPyVAN should be considered as a cause of sustained elevation in serum creatinine, new onset proteinuria, or new onset hematuria in kidney transplant recipients who have experienced an augmentation in immunosuppression such as patients undergoing chemotherapy for underlying malignancies.

## Introduction

Reactivation of BK Polyomavirus (BKPyV) is estimated to occur in over 20 % of kidney transplant recipients. While many cases are transient and/or respond to reduction in immunosuppression, persistent infection can result in BK Polyomavirus associated nephropathy (BKPyVAN) and premature graft loss [1]. Primary BKPyV infection occurs during childhood with 60–70 % of children showing seropositivity by age 10 [1], [2], [3]. Following the initial infection BKPyV remains dormant in the urothelium and renal tubules. In immunocompetent individuals the infection remains latent. However, reactivation of the virus may occur in the immunocompromised host. However, while immunosuppression is believed to be a risk factor for BKPyVAN, the rates of BKPyVAN in other immunosuppressed populations, including cancer patients receiving chemotherapy, is substantially lower compared to kidney transplant recipients [2], [4]. Reactivation of BKPyV first begins as low level BKPyV-viruria, with progression to BKPyV DNAemia, and finally BKPyVAN, a biopsy proven entity with tubulointersitial nephritis. In the kidney transplant recipient this must be distinguished from T cell mediated rejection as both processes can manifest with interstitial inflammation and tubulitis. The absence of vascular inflammation, BK PCR > 10,000 copies/mL in the blood, and a positive SV40-LTag immunohistochemistry staining that colocalizes with the tubulointerstitial inflammation are suggestive of BKPyVAN. The severity of BKPyVAN can further be classified via Banff ci-score or the American Society of Transplantation strata, both of which provide a semi-quantitative assessment of viral replication as greater viral replication is associated with increased risk of graft loss [5].

Most cases of BKPyVAN occur within 2 years of kidney transplantation as this is the period of time when cellular immunity is most suppressed due to induction immunosuppression. Herein is described a case of BKPyVAN precipitated by the administration of chemotherapy for the treatment of multiple myeloma in a kidney transplant recipient over a decade post transplantation.

## Case report

A female in her 60 s presented to transplant clinic with elevation in serum creatinine. She had received a deceased donor kidney transplant 15 years prior after becoming dialysis dependent from lupus nephritis.

She was initially diagnosed with lupus nephritis in her teens after presenting with arthralgias, malar rash, and hematuria. She received cyclophosphamide and responded to treatment with improvement in her systemic manifestations of lupus but unfortunately developed chronic kidney disease. Her renal function gradually deteriorated until she was started on renal replacement therapy in her 30 s. She received a deceased donor kidney transplant 9 years later.

She received basiliximab for induction and was maintained on prednisone, tacrolimus, and mycophenolate mofetil. Her initial kidney transplant course was uncomplicated for 12 years. She maintained a baseline creatinine ranged between 1.1 and 1.3 mg/dL and did not experience episodes of rejection, malignancy or opportunistic infections. Annual surveillance viral screening for BK was negative for 7 years post-transplant before testing was stopped. Twelve years post-transplant she was noted to have an elevated total serum protein level and serum immunofixation demonstrated an M spike; she was diagnosed with monoclonal gammopathy of unclear significance (MGUS) with the recommendation for surveillance monitoring. Routine surveillance showed stable M-spike until 18 months later when her levels increased. A bone marrow biopsy was performed and revealed 15 % plasma cells leading to a diagnosis of multiple myeloma (MM). Mycophenolate mofetil (MMF) was discontinued and she was started on bortezomib, lenalidomide, and dexamethasone for myeloma treatment. Her renal function remained stable throughout this period.

**Fig. 1:**
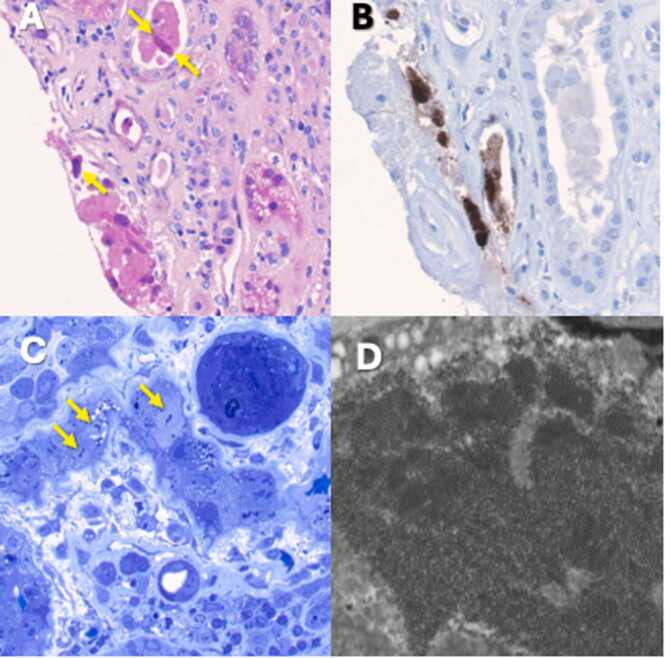
Transplant Kidney Biopsy A: Light microscopy reveals shedding of infected tubular epithelial cells into tubular lumina. There is severe viral cytopathic effect with nuclear enlargement, prominent nucleoli, and nuclear viral inclusions (arrows), hematoxylin and eosin stain, magnification 400X. B: Immunohistochemistry for polyomavirus infection is performed with a cross-reactive antibody against SV40 and reveals strong nuclear staining in infected tubular epithelial cells, magnification 400X. C: A toluidine blue stained section (magnification 400X) reveals severe viral cytopathic effect with nuclear enlargement with prominent nucleoli (arrow), and smudgy nuclear viral inclusions. D: Electron microscopy reveals the presence of viral particles measuring form 40–50 nm in diameter, focally arranged into lattice like structures within infected tubular epithelial cell nuclei (magnification 15,000X).

During her initial treatment for MM she developed anemia and acute kidney injury with an increase in serum creatinine to 1.6 mg/dL from 1.3 mg/dL. Carfilzomib was added to her multiple myeloma treatment regimen due to concern for refractory disease given development of new symptoms while on myeloma treatment. Her renal function stabilized around a serum creatinine 1.6 mg/dL and blood testing demonstrated evidence of serological remission with a loss of her M-spike following 9 months of treatment. On routine testing she was found to have an elevated serum creatinine of 4.26 mg/dL and was referred back to transplant clinic for evaluation. Urinalysis noted absence of proteinuria with a urine protein creatinine ratio of 0.04 g/g. She was referred for percutaneous kidney transplant biopsy due to rise in serum creatinine. Light microscopy revealed a patchy and dense interstitial inflammatory infiltrate composed primarily of lymphocytes and mature plasma cells. The inflammatory infiltrate was multifocally associated with foci of severe tubulitis, with focal tubular basement membrane rupture. The nuclei of these inflamed tubules focally showed severe viral cytopathic effect, with nuclear enlargement, irregular contours, prominent nucleoli, and smudgy "ground glass" viral inclusions. Immunohistochemical staining for polyomavirus was performed (with a cross reactive antibody against SV40) and revealed strong nuclear positivity in tubular epithelial cells, in a distribution which coincided with the foci of severe tubulointerstitial inflammation, consistent with polyomavirus nephropathy (Fig. 1). Importantly, there was no significant interstitial inflammation or tubulitis in areas lacking SV40 positive cells, and there was no intimal arteritis, arguing against the presence of T-cell mediated rejection. There was no significant microvascular inflammation, arguing against the presence of acute antibody mediated rejection. Electron microscopy revealed the presence of viral particles in few infected tubular epithelial cells. The particles measured approximately 40–50 nm in diameter and focally formed organized and tightly packed lattice-like arrays (Fig. 1). Following the pathology findings, serum BK viral PCR was sent and resulted as 236,000 copies/mL.

As she was already on reduced immunosuppression and being actively treated for myeloma, no additional therapy was given. She completed her chemotherapy 4 months later and a repeat bone marrow biopsy showed remission with < 1 % plasma cells. Unfortunately, her renal allograft function continued to deteriorate, and she resumed dialysis 8 months after diagnosis of BKPyVAN. Patient’s DNAemia was also not serially monitored after initial diagnosis as it was felt there would no change in management based on serum PCR levels.

## Discussion

BKPyVAN in kidney transplant recipients beyond 2 years is uncommon when patients are typically on maintenance immunosuppression regimens. In kidney transplant patients receiving additional immunosuppressive therapy, either in the form of treatment for rejection or with the administration of chemotherapy for an underlying malignancy, screening for BKPyV should be considered. As seen in this case late detection resulted in irreversible renal allograft injury and allograft loss.

Screening is generally recommended for the first 2–3 years post-transplant, coinciding with the highest risk of reactivation. The 2024 s International Consensus Guidelines on the Management of BK Polyomavirus in Kidney recommended all kidney transplant recipients be screened for serum BK DNAemia monthly before transitioning to every 3 months at month 9 until 2 years post-transplant [5]. Risk of BK occurring late post-transplant is considered low because immunosuppression dosing tends to be minimized and the effects of induction medications are no longer present. Screening beyond 2–3 years is not generally recommended. However, intensification of immunosuppression may result in BK reactivation, especially when ATG is used to treat allograft rejection [2], [6]. Screening after administration immunosuppression exposure may be indicated in these settings.

In general, BK DNAemia in native kidneys is a rare occurrence in the setting of immunosuppression [6]. Even in other solid organ transplantation, when similar immunosuppressive medication drugs are used, BKPyV and BKPyVAN rates are considerably lower than in kidney transplant recipients [6]. MM is considered to be a state of immunosuppression with an increased risk of bacterial and viral infections, especially during treatment. However, BKPyVAN in MM patients treated with chemotherapy has only been reported at the case report level, suggesting that there is an inherent process in kidney transplantation leading to increased risk for BK reactivation. In the largest reported cohort of post kidney transplant recipients who developed MM, Kornmann et al. did not report any cases of BKPyV [7]. Patients in this study only had reduction in immunosuppression following infection while receiving chemotherapy.

In this patient, BK screening did not reveal reactivation in the first 7 years and screening was stopped. Her renal function remained excellent for over a decade, arguing that reactivation of BKPyV, and the development of BKPyVAN, was triggered following treatment for MM. Interestingly, her MMF was discontinued prior to initiation of chemotherapy and her tacrolimus trough was maintained around 5 ng/mL in the setting of active malignancy. Other possible causes of kidney injury in this MM patient were excluded by biopsy, but included: rejection, light chain case nephropathy (myeloma kidney), amyloidosis, and acute tubular necrosis. The absence of significant proteinuria at time of biopsy argued against MM-related causes of kidney injury.

Reduction/cessation of anti-metabolite is commonly the first step in the management of BKPyVAN [8], [5]. Reduction in tacrolimus may be performed in non-responding patients, but does carry an increased risk of rejection [1], [5]. Given her underlying malignancy and need for adequate renal function for ongoing chemotherapy, her anti-metabolite was held. Other reported treatments for BKPyVAN have shown limited efficacy, including IVIG, leflunomide, fluroquinolone antibiotics, and the anti-viral cidofovir [1], [8], [5]. Emerging therapies, including BK-specific antibody and T cells, are currently in clinical trials.

She was re-evaluated for kidney transplantation and relisted, given remission of her myeloma and no active BKPyV.

## Conclusion

Late onset of BKVAN should be considered as a cause of kidney injury in kidney transplant recipients undergoing chemotherapy for underlying malignancies.

## CRediT authorship contribution statement

**Pryce Gaynor:** Writing – review & editing, Writing – original draft. **Erik L Lum:** Writing – review & editing, Data curation, Conceptualization. **Abhirami Shankar:** Writing – review & editing. **Jean Hou:** Writing – review & editing, Data curation. **Tram Higgins:** Writing – review & editing, Writing – original draft, Data curation. **Anum Hamiduzzaman:** Writing – original draft, Data curation, Conceptualization.

## Consent

Written informed consent was obtained from the patient for publication of this case report and accompanying images. A copy of the written consent is available for review by the Editor-in-Chief of this journal on request

## Ethical approval

Yes

## Funding

None.

## Declaration of Competing Interest

The authors declare that they have no known competing financial interests or personal relationships that could have appeared to influence the work reported in this paper.
